# Special Issue “Gastrointestinal Microbiota and Gut Barrier Impact Human Health and Disease”: Editorial

**DOI:** 10.3390/microorganisms11040985

**Published:** 2023-04-10

**Authors:** Pauline Raoul, Marco Cintoni, Emanuele Rinninella, Maria Cristina Mele

**Affiliations:** 1 UOC di Nutrizione Clinica, Dipartimento di Scienze Mediche e Chirurgiche Endocrino-Metaboliche, Fondazione Policlinico Universitario A. Gemelli IRCCS, Largo A. Gemelli 8, 00168 Rome, Italy; 2 Dipartimento di Medicina e Chirurgia Traslazionale, Università Cattolica Del Sacro Cuore, Largo F. Vito 1, 00168 Rome, Italy

The increasing incidence of non-communicable diseases is a worldwide public health issue, and the role of gut microbiota is becoming evident. Gut microbiota modifications due to various environmental factors are associated with impaired gut barrier integrity, increasing bacterial translocation and contributing to low-grade inflammation. Numerous recent studies have shown significant associations of the alterations of gut barrier integrity and gut microbiota changes with inflammation. Hence, understanding the triggering mechanisms of the inflammatory responses in patients affected by chronic diseases could enable the development of intervention strategies, such as personalized diet, prebiotics, probiotics, and fecal microbiota transplantation. This Special Issue on “Gastrointestinal Microbiota and Gut Barrier Impact Human Health and Disease” aimed to dissect the gut microbiota/gut barrier interactions, outlining their potential role in health and diseases. It compiles fifteen original papers and nine reviews.

Three original papers and one review published in this Special Issue focus on the modulation of gut microbiota in early life and even during pregnancy [1,2,3,4]. De Siena et al. highlight the strong relationship between dysbiosis and gestational diabetes mellitus, preterm birth, and pre-eclampsia [3]. Prolonged antibiotic therapy increases complications, such as necrotizing enterocolitis in preterm infants. Chabaan et al. show in a neonatal mice model that antibiotic treatment altered gut development by decreasing villi height and intestinal cell proliferation, such as goblet and Paneth cells [1]. A prospective longitudinal study [2] demonstrates that the gut microbiome is altered in pediatric abdominal pain disorders compared to controls. In particular, the *Faecalibacterium/Bacteroides* ratio could potentially be used as a biomarker for diagnosing functional abdominal pain disorders. The role of the Proteobacteria family in pediatric patients has also been studied, especially in regard to the pathophysiology of obesity [4,5]. Indeed, in the study of Orsso et al., the fecal samples of children with insulin resistance and obesity presented a lower abundance of bacteria related to butyrate production and a lower α-diversity for Proteobacteria species compared with those of healthy children. The higher homeostatic model assessment for insulin resistance levels (HOMA) has also been associated with fewer amino acids and short chain fatty acids (SCFAs) biosynthesis pathways. Specifically, a systematic review of ten original studies [5] outlines the critical role of the Proteobacteria *Akkermansia muciniphila* in improving insulin sensitivity and regulating metabolism. Pharmacological and nutritional interventions by modulating the abundance of *A. muciniphila* could become a gold standard for preventing or treating obesity and its comorbidities [6].

Numerous studies in this Special Issue discuss the ways in which interactions between the host and the gut microbiota can alter clinical outcomes of various non-communicable diseases, such as autism spectrum disorders, ischemia-reperfusion, and kidney diseases. Interestingly, a translational mice study demonstrates the ability of fecal supernatant from autism spectrum disorders individuals to induce gastrointestinal and enteric nervous system alterations. The authors assess the ways in which gut microbiota could contribute to pathophysiological mechanisms underlying autism spectrum disorders-associated gastrointestinal symptoms [7]. Another mice model study [8] shows a protective role of altered Schaedler flora against the ischemia-reperfusion-induced increase in leukocyte adherence to the injured endothelium of mesenteric venules. Gut dysbiosis may also induce chronic inflammation in patients with chronic kidney disease. The phosphate binder treatment can alter gut microbiota composition in such patients. A human study by Wu et al. analyses the composition of fecal samples in hemodialysis patients treated with calcium carbonate or ferric citrate [9]. The authors conclude that calcium carbonate treatment results in a lower microbial diversity than ferric citrate treatment. Thus, as the study by Souai et al. also suggests, the analysis of the gut microbial composition may evaluate the effects of drugs such as those prescribed in organ transplantations [10]. Indeed, there is a significant association between gut microbiota composition and the clinical conditions of renal transplant recipients. Dysbiosis is also associated with respiratory viral infections through the gut–lung axis [11]. A prospective cross-sectional study [11] shows a significant association between the severity of COVID-19 and the decrease in abundance of *Prevotella*, *F. prausnitzii*, and *B. Plebius*, as well as the increase in Bacteroidetes. Thus, gut dysbiosis would represent a predisposing factor for COVID-19 pathophysiology.

The gut microbiota composition also represents a feature of inflammatory bowel disease (IBD), the etiology of which is still poorly understood. Specifically, Baldelli et al. highlight the close association of the IBD pathogenesis with gut dysbiosis, specifically with the increased abundance of *Enterobacteriaceae* [12], including *Escherichia coli* (*E. Coli*), one of the first species colonizing the gut in the first years of infancy. Two original studies in this Special Issue focus on the diversity and adaptations of *E. coli* strains in IBD [13,14]. Siniagina et al. highlight the high capacity of these strains to colonize the gut [13]. Interestingly, the hemolysin-producing *E. coli* strain represents an essential factor in the pathogenesis of ulcerative colitis by altering the gut epithelial barrier [14]. In this setting, the disruption of gut microbiota provides an ideal environment for infections such as *Clostridioides difficile* infection (CDI). Sanchez et al. show the significantly different gut microbiota in patients with CDI compared with controls in terms of richness and diversity [15]. *Clostridioides difficile* exacerbates IBD, while IBD represents a risk factor for *Clostridioides difficile* [16].

The inflammation/immunity–microbiota axis is also evaluated by Abdalqadir et al. [17]. A review highlights the role of glucagon-like peptides, intestinal hormones that regulate glycemia by stimulating insulin production, in the intestine–microbiota–immune system interplay. These peptides can preserve metabolic homeostasis and gut barrier integrity [17]. Other metabolites studied are indole and indole-related compounds [18]. The indole-derived co-metabolite isatin could accumulate in the brain, positively or negatively modulating brain functions, depending on the doses used. In experimental studies, oxindole, another bacterial metabolite, has a negative effect on the central nervous system, while indoxyl sulfate can exert positive or detrimental effects depending on the dose used.

In recent years, the modulation of gut microbiota through prebiotics, probiotics, and fecal microbiota transplant has garnered interest to alleviate intestinal barrier dysfunctions. Different interventions have been studied in this Special Issue. As a prebiotic effect, Saxami et al. show that β-glucan *P. Eryngii* mushrooms [19] could have positive and potential protective effects on intestinal barrier integrity. Probiotics are live microorganisms that, when administered in adequate amounts, could also benefit the host. Research into yeasts, such as cheese yeasts, shows potentially beneficial influences on human gut health and preventive effects against microbial pathogens [20]. To date, the current knowledge around the gut microbiota response to short-term and long-term dietary interventions is growing with the aim of identifying the microbiota response to diet precisely. The beneficial effects of polyphenols on intestinal health, particularly intestinal barrier integrity, have been investigated in in vitro conditions [21]. Dietary restrictions are also interesting strategies for preventing and treating non-communicable diseases through the restoration of the microbiota [22]. On the other hand, many artificial food additives could exacerbate chronic gut inflammation through gut microbiota compositional alterations and gut barrier impairment, especially in patients with IBD predisposition [23]. 

It is clear from the papers published in this Special Issue that the field of gut microbiota–gut barrier–health and disease interplay has many avenues to explore. Several lines of research have demonstrated the importance of early life microbiota and diet in developing a core gut microbiota and a balanced immune system. Over the course of life, structural and functional dysbiosis could be induced by a wide range of environmental factors. Gut dysbiosis leads to the development of different pathologies, such as obesity, insulin resistance, IBDs, cancer, and autism. Gut microbiota and gut barrier integrity bridge many, even distant, host zones through microbial metabolite production and the immune system. Most metagenomic studies explore microbiome variations from healthy ones to those with dysbiosis. For instance, the study by Villanova et al. describes the fully sequenced genomes of new variants of squash mosaic viruses found in fecal human samples and analyzes the viral profile by metagenomics in the context of diarrhea symptomatology [24].

We look forward to more studies focusing on gut dysbiosis, gut barrier permeability, and gut bacterial metabolites to clarify the mechanisms of interactions between microorganisms and molecular patterns. We hope that the clinical application of gut microbiota modulation therapies, such as personalized medicine (including diet), can improve the clinical outcomes of patients.
